# Minerva: a light-weight, narrative image browser for multiplexed tissue images

**DOI:** 10.21105/joss.02579

**Published:** 2020-10-15

**Authors:** John Hoffer, Rumana Rashid, Jeremy L. Muhlich, Yu-An Chen, Douglas Peter William Russell, Juha Ruokonen, Robert Krueger, Hanspeter Pfister, Sandro Santagata, Peter K. Sorger

**Affiliations:** 1 Laboratory of Systems Pharmacology, Harvard Medical School, Boston, MA; 2 Ludwig Center for Cancer Research at Harvard, Harvard Medical School, Boston, MA; 3 Department of Pathology, Brigham and Women’s Hospital, Harvard Medical School, Boston, MA; 4 Mathworks, Natick, MA; 5 School of Engineering and Applied Sciences, Harvard University, Cambridge, MA; 6 Department of Systems Biology, Harvard Medical School, Boston, MA

## Abstract

Advances in highly multiplexed tissue imaging are transforming our understanding of human biology by enabling detection and localization of 10-100 proteins at subcellular resolution (Bodenmiller, 2016). Efforts are now underway to create public atlases of multiplexed images of normal and diseased tissues (Rozenblatt-Rosen et al., 2020). Both research and clinical applications of tissue imaging benefit from recording data from complete specimens so that data on cell state and composition can be studied in the context of overall tissue architecture. As a practical matter, specimen size is limited by the dimensions of microscopy slides (2.5 × 7.5 cm or ~2-8 cm^2^ of tissue depending on shape). With current microscopy technology, specimens of this size can be imaged at sub-micron resolution across ~60 spectral channels and ~10^6^ cells, resulting in image files of terabyte size. However, the rich detail and multiscale properties of these images pose a substantial computational challenge (Rashid et al., 2020). See Rashid et al. (2020) for an comparison of existing visualization tools targeting these multiplexed tissue images.

In this paper we describe a new open-source visualization tool, Minerva, which facilitates intuitive real-time exploration of large multiplexed images on the web. Minerva employs the OpenSeadragon (“OpenSeadragon,” n.d.) framework to render images at multiple resolutions and makes it possible to pan and zoom across across images in a process analogous to Google Maps. However, tissues contain many specialized structures recognizable by pathologists and histologists but not necessarily by many other scientific or medical users. To capitalize on specialized histology expertise we require software that mimics the current practice in which a pathologists sits alongside a colleague and reviews a specimen by moving from point to point and switching between high and low magnifications.

Minerva is designed to generate precisely these types of interactive guides or “stories”. The author of a story creates specific waypoints in the image each with a text description, position, zoom level, and overlaid shape annotations. In the case of highly multiplexed images, a subset of channels is chosen for display at each waypoint (typically 4-8 superimposed channels). Authors also add interactive single-cell data scatterplots, bar charts, heatmaps, and cell outlines with two-way linked navigation between the plots and points in the image. Minerva is deployed simply and inexpensively via static web hosting. See Figure 1 for schematic of the workflow and system components.

Minerva is not designed to solve all analytical and visualization problems encountered in multiplexed tissue imaging. Instead, it is a publication tool specialized to the task of making data shareable and broadly intelligible without requiring specialized software on the user side. As such, Minerva is designed to be one component in an ecosystem of interoperable, open-source software tools.

Minerva comprises two components, *Minerva Story* and *Minerva Author. Minerva Story* is a single-page web application for presenting an image and its narrative to end users. OpenSeadragon (“OpenSeadragon,” n.d.) is used to render tiled JPEG image pyramids overlaid with the author’s narrative text, graphical annotations and data plots. Audio presentation of the narrative text is optionally provided through integration with Amazon’s Polly text-to-speech service. The image pyramid URL and all narrative details are loaded from an independent JSON story definition file. Minerva Story can be hosted through GitHub Pages or any other web host supporting static content such as Amazon S3.

*Minerva Author* is a desktop application for constructing narratives (stories) for Minerva Story. It is a JavaScript React web application with a Python Flask backend, packaged with Pylnstaller as a native application. To create a narrative, the author first imports an image in standard OME-TIFF pyramid or SVS formats. Both RGB images (brightfield, H&E, immunohistochemistry, etc.) and multi-channel fluorescence images (immunofluorescence, CODEX, CyCIF, etc.) are supported. Fluorescence image rendering can be controlled through per-channel contrast adjustment and pseudocoloring. The author then adds one or more story waypoints. For each waypoint, the author can type a text description, draw polygon or arrow annotations to highlight specific cells, regions, and histological features, and choose specific image channels to present. See Figure 2 for the Minerva Author interface. After the waypoints are complete, Minerva Author renders the input image into one or more RGB JPEG image pyramids and produces a JSON story definition file. Finally an author uploads the JPEG images (vastly smaller than the raw image data) and JSON file to a web host along with the Minerva Story HTML files. Story waypoints can be also augmented with interactive linked data visualizations. Adding data visualization currently requires manually editing the JSON file but a version of Minerva Author is in development for adding these types of visualizations natively.

Minerva offers two approaches to exploring a narrative. First, in the author-driven approach, users can progress through a story in a linear path using forward and back navigation buttons, allowing an efficient introduction to and expert overview of the data. Second, in a free-exploration approach, the user is free to move to any position or zoom level and select any channel grouping or segmentation mask. Users can also take a hybrid approach by following a story and then departing from it to freely explore or skip between waypoints. By returning to a story waypoint the narrated overview can be resumed, much as one follows an audio guide in a museum. Minerva supports creating deep links directly to any position and zoom level in an image simply by copying and sharing the current URL from the browser. Users can optionally write a text note and draw a custom shape annotation that is automatically presented to the recipients.

We have identified multiple applications for Minerva: visualizing cell-type classifiers in image space, validating results of unsupervised clustering of single-cell data, manual scanning for spatial patterns, assessing quality of antibody staining, obtaining second opinions from collaborators, sharing high-resolution primary image data alongside published manuscripts, and creating educational content for medical trainees. Minerva is also being used by national consortia to build tissue atlases, and we plan to add it to existing genome browsers such as cBioPortal (Cerami et al., 2012) and thereby facilitate joint exploration of genomic and histological data.

Detailed documentation with step-by-step instructions for using Minerva, tutorial videos, exemplar data, and details on software testing are located alongside the source code on the Minerva wiki on Github. A wide variety of exemplary Minerva stories can be found at https://www.cycif.org/software/minerva.

## Figures and Tables

**Figure 1:**
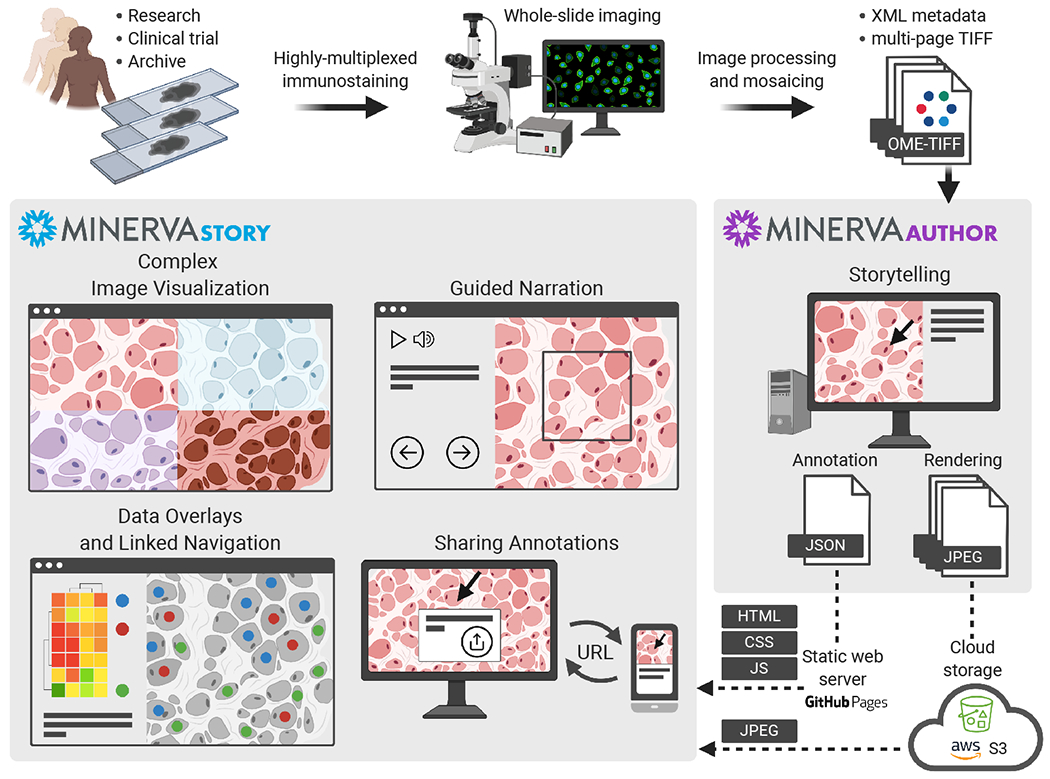
Minerva Workflow.

**Figure 2:**
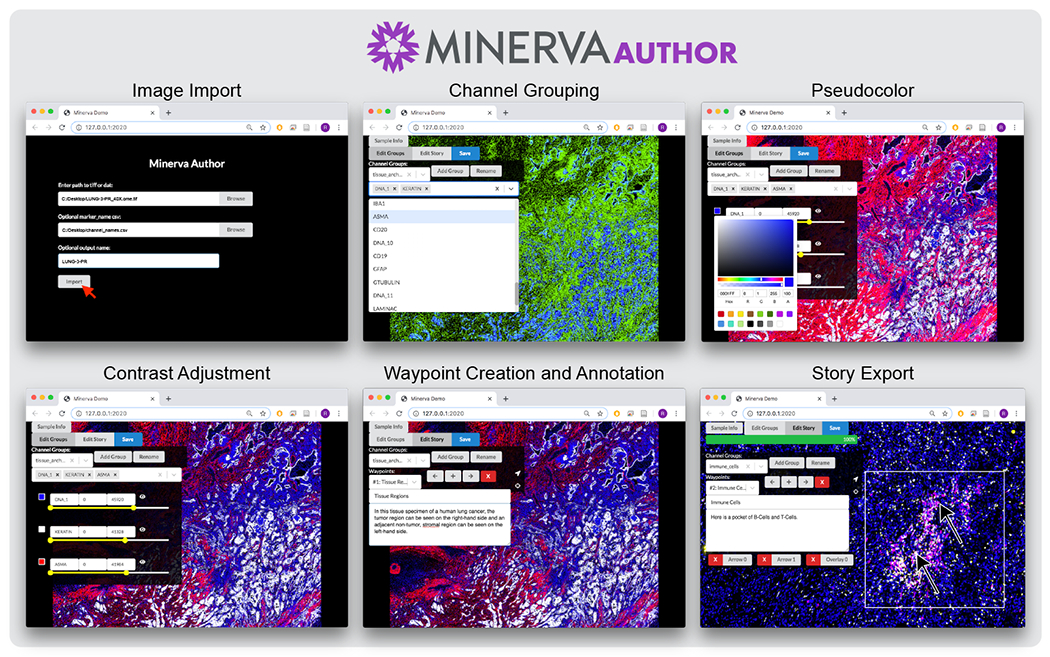
Minerva Author Interface.
